# Auditory dysfunction associated with solvent exposure

**DOI:** 10.1186/1471-2458-13-39

**Published:** 2013-01-16

**Authors:** Adrian Fuente, Bradley McPherson, Louise Hickson

**Affiliations:** 1 School of Health and Rehabilitation Sciences. The University of Queensland, Room 825, Level 8, Therapies Building (No 84A), St Lucia, Brisbane, QLD 4072, Australia; 2 Centre for Communication Disorders. The University of Hong Kong, 5/F Prince Philip Dental Hospital, 34 Hospital Road, Sai Ying Pun, Hong Kong, China

**Keywords:** Assessment, Hearing loss, Solvents, Temporal resolution

## Abstract

**Background:**

A number of studies have demonstrated that solvents may induce auditory dysfunction. However, there is still little knowledge regarding the main signs and symptoms of solvent-induced hearing loss (SIHL). The aim of this research was to investigate the association between solvent exposure and adverse effects on peripheral and central auditory functioning with a comprehensive audiological test battery.

**Methods:**

Seventy-two solvent-exposed workers and 72 non-exposed workers were selected to participate in the study. The test battery comprised pure-tone audiometry (PTA), transient evoked otoacoustic emissions (TEOAE), Random Gap Detection (RGD) and Hearing-in-Noise test (HINT).

**Results:**

Solvent-exposed subjects presented with poorer mean test results than non-exposed subjects. A bivariate and multivariate linear regression model analysis was performed. One model for each auditory outcome (PTA, TEOAE, RGD and HINT) was independently constructed. For all of the models solvent exposure was significantly associated with the auditory outcome. Age also appeared significantly associated with some auditory outcomes.

**Conclusions:**

This study provides further evidence of the possible adverse effect of solvents on the peripheral and central auditory functioning. A discussion of these effects and the utility of selected hearing tests to assess SIHL is addressed.

## Background

A solvent is a liquid or solid phase containing more than one substance [1]. Most solvents are colourless liquids at room temperature that volatise easily and have strong odours. Solvents are most commonly inhaled in their volatised form and absorbed through the respiratory tract. Organic solvents are widely used around the world and many different industrial processes require their use. Millions of persons are currently exposed to solvents in their workplaces [2], mostly in developing countries. One of the first reports of the adverse effects of solvents on human hearing came from Szulck-Kuberska et al. [3] who studied a group of workers exposed to trichloroethylene. Current scientific evidence from animal models indicates that solvents such as toluene, styrene, xylene, n-hexane, and ethyl benzene, as well as trichloroethylene, have ototoxic effects. The cochlear damage induced by solvents starts from the third row of outer hair cells (OHC) and then, if the exposure continues, the damage progresses to the second and first row of OHC [4,5]. The mid-frequency region of the cochlea of rats is particularly affected by these chemicals [4,6]. Human cross-sectional studies have shown that workers exposed to solvents exhibit a higher prevalence of hearing loss in comparison to non-exposed control subjects [7-9]. Other human studies suggest adverse central auditory effects associated with solvent exposure [10,11].

Despite the large number of studies on solvent-induced hearing loss (SIHL) in both animals and humans the current knowledge of the signs and symptoms of SIHL in humans is limited. It is suggested from animal models that SIHL is associated with OHC dysfunction. However, from human studies there is still limited evidence concerning cochlear dysfunction associated with SIHL. Sulkowski et al. [12] found lower amplitudes for transient evoked otoacoustic emissions (TEOAEs) and distortion product otoacoustic emissions (DPOAEs) among solvent-exposed subjects in comparison to non-exposed control subjects. Johnson et al. [13] found significant differences between solvent-exposed and control subjects for the input/output function of the DPOAE only at lower intensities. However, no differences for TEOAE and DPOAE amplitudes were observed between groups in that study. Thus, further research is needed to determine OHC dysfunction in human subjects exposed to solvents. Lower amplitudes for TEOAEs or DPOAEs may be the key for the early detection of SIHL.

Most of the studies conducted in human populations exposed to solvents have mainly investigated audiometric pure-tone thresholds. Some studies have further investigated the central auditory nervous system (CANS) [11,13-18] and the results suggest CANS dysfunction is associated with solvent exposure. CANS dysfunction is likely to affect speech perception in noise. However, there is scant knowledge regarding the possible difficulties solvent-exposed subjects may encounter understanding speech in the presence of background noise. There is little evidence as well in terms of which aspects of the function of the CANS are adversely affected by solvent exposure.

In summary, most of the human investigations on SIHL have used pure-tone audiometric results as the hearing outcome studied. Only a small number of studies have further investigated the CANS in human subjects exposed to solvents. Still fewer studies have investigated both OHC and the CANS in the same samples of solvent-exposed subjects. The present study aimed to obtain reliable, objective data on other expressions of SIHL beyond pure-tone thresholds, such as potential central auditory dysfunction and speech-in-noise difficulties. The other important issue is the possible added value of OAE measurements, which are suggested to be more sensitive and predictive of cochlear dysfunction induced by ototoxic agents. For the aforementioned research aims the use of a comprehensive audiological test battery was devised.

## Methods

### Study design

This is a cross-sectional study of subjects exposed to solvents (study group) and subjects without solvent exposure (control group). The data presented in this article came from a larger study investigating the oto-and neuro-toxic effects of solvents.

### Ethical approval

All research procedures were approved prior the commencement of the study by the Ethics Committee of the Faculty of Medicine, University of Chile.

### Study participants and data collection

A non-probability, convenience sampling technique was used to select workers exposed to solvents. Two paint making factories from Santiago, Chile, gave access to their facilities. Initial inclusion criteria included (a) solvent exposure for at least 1 year, (b) age between 18 and 55 years, (c) noise exposure below 85 dBA in the workplace, and (d) job categories where workers were directly exposed to solvents. These job categories included maintenance engineers, production supervisors, machine operators, quality control employers, helpers, mixers, and hazardous waste handlers. For each solvent-exposed subject who was eligible (see further selection criteria below) to be included in the final sample, an educational level-matched non-exposed control subject was selected. Educational level was defined as incomplete secondary education, complete secondary education or tertiary studies. Non-exposed control group subjects were non-academic personnel of the University of Chile who were recruited through public advertisements and by word-of-mouth.

Each participant (from both groups) was individually scheduled for a single 150-minute appointment at the audiology laboratory, Faculty of Medicine, University of Chile. The session was conducted by the first author who is a trained audiologist. The assessment session started with an oral explanation about the research aims and procedures to be used. Subjects were invited to ask for clarification in case something was not clear. Also, an informed consent form was provided. Subjects were asked to read the form and sign it if they agree to proceed with the interview and auditory assessment. None of the subjects refused to proceed. Once the research aims and assessment procedures were clear an interview based on a questionnaire adapted from the Noisechem questionnaire [19] was carried out in both solvent-exposed and control group subjects. This questionnaire addressed subject ear history and medical conditions that may be associated with the onset of auditory dysfunction; occupational history (e.g., previous jobs exposed to noise, use of solvents, use of hearing and respiratory protection at work, and tenure at each workplace); and non-occupational noise exposure. The questionnaire was utilised for two main reasons, (1) to select only subjects with an absence of variables that may be related to auditory dysfunction, and (2) to explore covariates such as smoking, alcohol consumption, years working at the factory exposed to solvents, and previous noise exposure for further inclusion in multivariate analyses. Based on the questionnaire data subjects were excluded from the final sample if they presented with one or more of the following variables (a) history of middle ear infections, (b) treatment with ototoxic drugs, and (c) medical conditions such as diabetes, metabolic dysfunction, past head trauma, neurological disorders and kidney failure. Following the interview, bilateral otoscopy and tympanometry were carried out. Only subjects with an absence of visible pathologic alteration of the ear canal and normal type A tympanometric results (tympanic peak pressure between −100 and +50 daPa and static compliance ≥ 0.2 mL) [20] were included in the sample. All selected subjects within the same session were then evaluated with a comprehensive battery of hearing tests investigating pure-tone thresholds, OAEs, CANS through a temporal processing task and speech perception in quiet and in noise. The order of testing was the same for all subjects.

### Workplace environment

The main task of both factories was the manufacture of paints. Methyl ethyl ketone (MEK), toluene, xylene and Stoddard solvent (mineral spirits) were used in major quantities but many other solvents including benzol, esters and alcohols were also regularly used in the factories. Previous records of airborne solvent concentrations were available from both factories, and the mean concentrations are shown in Table 1. The mean airborne concentrations for MEK, toluene, xylene and Stoddard solvent were calculated based on 15 single measurements of airborne solvent vapour concentrations which were available from both factories. Tubes of activated carbon were used to obtained air samples in a total of 7 workstations between 2004 and 2007. The components used in both paint-making factories are mixed in different concentrations depending on the desired production outcome. Workers were therefore exposed to different mixtures of solvents over time. Solvents were used in the following processes: a) at the beginning of the process when the pigment is premixed with resin, one or more solvents are added to form a paste; b) the paste mixture is then taken to a sand-mill or in a high-speed dispersion tank to disperse the premixed paste; c) the paste must then be thinned to produce the final product by agitation in large kettles with specific amounts/types of solvents according to the desired product; d) the finished paint is then pumped into the canning room. Waste containing solvents is also generated during the production process. Specific personnel handle the waste created, and solvents are also used for cleaning purposes.

**Table 1:** Means, standard deviation, and range of airborne concentrations for methyl ethyl ketone (MEK), toluene, xylene and Stoddard solvent for the workplaces of the selected subjects from both paint making factories

**Solvent**	**Mean (S.D)**	**Range**
MEK (n=15)	8.68 (11.39)	0.01- 32.5
Toluene (n=15)	13.69 (25.53)	0.01- 114.8
Xylene (n=15)	26.48 (41.86)	0.2- 173.1
Stoddard solvent (=15)	111.91 (236.63)	0.01- 1300

n= number of records.

Values are mg/m3.

### Audiological assessment

#### Audiometric thresholds

Pure-tone air-and bone-conduction thresholds were obtained using an Interacoustics AC33 clinical audiometer with TDH-39P headphones (calibrated according to ISO 389 series). Pure-tone measurements were all conducted in a double-walled sound treated room meeting ISO 8253–1 standards of ambient sound pressure levels. Air conduction pure-tone thresholds from 250 to 8000 Hz were obtained. The presentation order was as follows: 1000, 2000, 3000, 4000, 6000, 8000, 500, and 250 Hz. The modified Hughson and Westlake [21] procedure described by Carhart and Jerger [22] was used to obtain the hearing thresholds. Also, bone-conduction pure-tone thresholds from 500 to 4000 Hz were obtained. The presentation order was as follows: 1000, 2000, 3000, 4000, and 500 Hz. Stimuli were delivered to each mastoid through a Radioear B-71 bone vibrator. The same procedure as for air-conduction thresholds was utilised to obtain the bone conduction hearing thresholds. Subjects with a conductive component in the audiogram (presence of an air-bone gap at two or more frequencies equal or higher than 10 dB HL) were excluded from the final sample. For the purpose of the statistical analysis, the binaural average of pure-tone thresholds for both ears at 500, 1000, 2000, 3000, 4000, 6000 and 8000 Hz (PTA) was used. The binaural average of pure-tone thresholds (PTA) is expressed by the following equation: [(right ear threshold at 500 + 1000 + 2000 + 3000 + 4000 + 6000 + 8000 Hz) + (left ear threshold at 500 + 1000 + 2000 + 3000 + 4000 + 6000 + 8000 Hz)]/14].

### Transient evoked otoacoustic emissions (TEOAEs)

A portable Echoport plus (Otodynamics, London, calibrated according to manufacturer recommendations) was utilised for TEOAEs. This equipment was connected to a desktop computer which had ILO 88 OAE analysis software. Stimuli were delivered to the ears via an adult B type ILO otoacoustic emissions probe [23]. The evoked stimuli used were 80 μs rectangular clicks presented at 80 ± 2 dB peSPL. Nonlinear click stimuli were used. The response time window was set at 2.5 – 20 ms and the band-pass filter was set in the range from 500 Hz to 5000 Hz. TEAOEs were analysed by deriving the mean response for each ear in dB SNR. Amplitudes at each frequency band were also examined. The binaural response of TEOAEs in dB SNR was then calculated and further used in the statistical analyses. The binaural average of the mean amplitude of TEOAEs is expressed by the following formula: (mean TEOAEs dB SNR right ear + mean TEOAEs dB SNR left ear)/2.

### Random Gap Detection test –a measure of the function of temporal processing within the CANS

This test was used to assess temporal resolution, which is an aspect of temporal processing within the CANS [24]. This procedure was carried out using the test compact disk [25] and a compact disk player (LG 7311 N) which was connected to the audiometer mentioned above. A 1000 Hz calibration tone recorded on the compact disk was used to determine output intensity. At 50 dB HL, stimuli comprising two tones that differed in their onset time were presented binaurally. Subjects were asked to state whether they heard one or two tones at each presentation. Thresholds in milliseconds for each frequency tested (500, 1000, 2000, and 4000 Hz) and for click stimuli were calculated. Thresholds were defined as the minimum difference in milliseconds between the onset of both stimuli that was detected by the subject. For the purpose of analysis, the binaural average of random gap detection thresholds at 500, 1000, 2000, and 4000 Hz, and clicks (RGD) was used. The binaural average of random gap detection thresholds is expressed by the following equation: (binaural gap detection threshold at 500 + 1000 + 2000 + 4000 Hz + clicks)/5.

### Speech discrimination in quiet and in noise

The Hearing-in-Noise Test—HINT [26] with Latin American Spanish sentence module— was utilised to investigate speech discrimination in quiet and in noise. Stimuli (sentences) and noise were delivered via TDH-39P headphones. The HINT system, including the headphones, was calibrated according to manufacturer recommendations. HINT headphones use digital filtering that simulates the head-related transfer functions that would occur in the sound field. To calculate the speech reception threshold (SRT) in quiet a set of 20 sentences were presented binaurally (HINT SRT). Subjects were asked to repeat each sentence heard. The sentences were presented using an adaptive procedure where the sentence levels are varied according to the accuracy of the listener’s responses. Then, to assess speech discrimination in noise, signal-to-noise ratios (SNRs) required for 50% speech discrimination were calculated for different noise and sentence conditions. The HINT noise is spectrally matched to the average long term spectrum of the sentences. Therefore, on average the SNR is approximately equal to all frequencies [26]. The SNRs are obtained with an adaptive psychophysical procedure. Three sentence-in-noise conditions were tested with HINT. First, a set of 20 sentences and the masking noise were simultaneously presented from the same location (in front of the subject), that is, no spatial separation between the noise and the sentences (HINT 1). Then, noise was presented to the right ear and a new set of 20 sentences were simultaneously presented to the front (HINT 2). The third condition implied the presentation of noise to the left ear and a new set of 20 sentences simultaneously presented to the front (HINT 3). Finally, a composite score was calculated by combining the results of HINT 1, HINT 2, and HINT 3 (HINT composite). For the purpose of analysis, the binaural speech discrimination in quiet score (HINT SRT) and binaural speech discrimination in noise score (HINT composite) were used.

### Data analysis

Considering that age is an important covariate for hearing outcomes a Mann–Whitney test was computed in order to test for possible age differences between solvent-exposed and non-exposed subjects. Significant differences were considered at α < 0.05.

Audiometric pure-tone thresholds for each ear at each frequency (250–8000 Hz) were compared between solvent-exposed and non-exposed control subjects using Mann–Whitney tests with a Bonferroni adjustment of the p-value due to multiple comparisons of the same hearing outcome. The total number of comparisons was 16, thus the adjusted p-value for significance was 0.003. Mann–Whitney tests were also computed to investigate possible significant differences between solvent-exposed and non-exposed control subjects for the hearing outcomes of TEOAEs, RGD, HINT SRT and HINT Composite. Significant differences were considered at α < 0.05.

Simple linear regression analyses were carried out to examine associations between hearing outcomes (PTA, TEOAEs, RGD, HINT SRT and HINT composite) and the continuous covariates of age, smoking (number of cigarettes per week), alcohol consumption (number of drinks per month), and number of years working at the factory exposed to solvents. All factors were considered to be continuous variables. In addition, the categorical variables of gender, presence or absence of previously occupational noise exposure, and presence or absence of shooting practice were compared to hearing outcomes using simple linear regression. Multiple linear regressions were then performed to separately model the association between each of the five hearing outcome measures and the risk factors for auditory dysfunction tested in the simple linear regression models. A backward elimination technique was used with each model to select those risk factors remaining significant in the adjusted analysis, using a selection criterion of α < 0.05. In order to investigate the possible confounding effect of age, the models included the interaction term between solvent exposure group (exposed versus non-exposed) and age. The 95 percent confidence level (α = 0.05) was used in all tests of significance for the simple and multiple linear regressions and all statistical analyses were carried out using SPSS 14.0.

## Results

Initially, a total of 83 solvent-exposed workers and 79 non-exposed control subjects attended the assessment session. Eleven solvent-exposed subjects and 7 non-exposed control subjects were excluded from the sample due to the presence of variables which were part of the exclusion criteria. Five solvent-exposed and 1 control group subject were excluded due to history of otitis media, 2 solvent-exposed subjects were excluded due to history of head trauma, 3 solvent-exposed subjects and 2 control-group subjects were excluded due to obstructive cerumen in their ear canals, 1 solvent-exposed subject and 4 non-exposed control subjects were excluded due to tympanometric results different than Type A and/or the presence of a conductive component in the audiogram. Thus, the final sample which was further studied comprised 72 solvent-exposed subjects, and 72 educational-level matched, non-exposed control subjects. Table 2 summarises the demographics and other characteristics of both groups. The group of solvent-exposed subjects was comprised of fewer females than the control group, and they also were slightly older than control group subjects.

**Table 2:** Mean, standard deviation, median, and range for the covariates for the total group and subgroups of subjects

**Variables**	**All (n=144)**			**Group 1 (n=72)**			**Group 2 (n=72)**		
	**Mean**	**Median**	**Range**	**Mean**	**Median**	**Range**	**Mean**	**Median**	**Range**
Age (years)	38.7 (7.8)	38.0	22-55	39.9 (8.5)	43.0	22-52	37.5 (7.1)	36.0	26-55
**Gender**									
Female	20			6			14		
Male	124			66			58		
PNE	30			27			3		
**Shooting**									
WP	3			3			0		
NP	39			33			6		
Tenure	-	-	-	15.8 (8.1)	16.5	2-34	0	0	0
Smoking	21.26 (30.7)	14.0	0-140	26.8 (32.1)	14.0	0-140	18.7 (15.8)	12	0-80
Alcohol	17.4 (17.8)	13.5	0-60	15.6 (16.3)	13.5	0-60	31.2 (24.4)	27	0-60

*Note*. Group 1: Solvent-exposed workers Group 2: Non-exposed workers.

() Standard deviation.

Alcohol. Number of drinks per month.

Smoking. Number of cigarettes per week.

Tenure. Years working at the factory exposed to solvents.

PNE. Number of subjects who have been exposed to noise in previous jobs.

Shooting WP. Number of subjects who have shot wearing hearing protection.

Shooting NP. Number of subjects who have shot without wearing hearing protection.

### Group comparisons

No significant age differences were found between the final samples of solvent-exposed (n=72) and non-exposed control (n=72) groups (Z= −1.72, P > 0.05).

**Figure 1:**
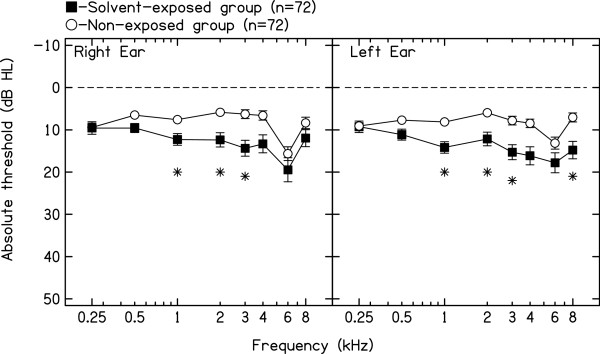
Mean right and left ear pure-tone thresholds (250–8000 Hz) and standard errors for both groups of subjects. * denotes significant differences between solvent-exposed and non-exposed control subjects at p < 0.003 (Mann–Whitney test).

Figure 1 shows the mean air conduction pure-tone thresholds (250–8000 Hz) for the right and left ears for solvent-exposed and non-exposed control subjects. Both groups of subjects presented with grand mean pure-tone hearing thresholds within the normal range (equal or better than 25 dB HL). However, solvent-exposed subjects presented with significantly (p < 0.003) worse pure-tone thresholds than non-exposed control subjects for 1000, 2000 and 3000 Hz for the right ear, and for 1000, 2000, 3000 and 8000 Hz for the left ear.

**Figure 2:**
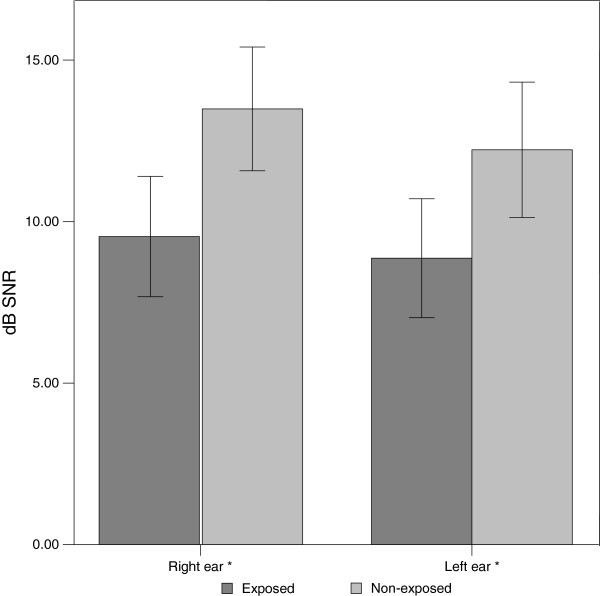
Means and standard error bars for TEOAE dB SNR in the right ear and left ear for solvent-exposed (n=72) and non-exposed (n=72) subjects. * denotes significant differences between solvent-exposed and non-exposed control subjects at p < 0.05 (Mann–Whitney test).

Figure 2 shows the mean dB SNR for TEOAEs for the right and left ears for both groups. Solvent-exposed subjects presented with significantly lower (p < 0.01) dB SNR than non-exposed control subjects for the TEAOEs in both ears. Table 3 shows the mean, SD, minimum and maximum for each group of subjects for RGD subtests and HINT subtests. Solvent-exposed subjects presented with significantly (p < 0.05) poorer results than non-exposed control subjects for all RGD subtests and for HINT SRT, HINT 1, HINT 2 and HINT composite. No significant (p > 0.05) differences between groups were observed for HINT 3.

**Table 3:** Mean, standard deviation, minimum and maximum for RGD, and HINT for both groups

**Solvent-exposed subjects (n=72)**					**Non-exposed subjects (n=72)**				**p-value (Mann–Whitney Test)**
	**Mean**	**S.D.**	**Min**	**Max**	**Mean**	**S.D.**	**Min**	**Max**	
**RGD 0.5**	12.46	10.65	2	60	8.16	6.94	2	25	p=0.013
**RGD 1**	14.46	10.38	2	60	7.02	4.25	2	15	P<0.0001
**RGD 2**	14.24	13.26	2	60	8.26	6.25	2	30	p=0.001
**RGD 4**	15.17	12.66	2	60	9.45	8.18	2	50	p=0.001
**RGD C**	10.34	7.57	2	40	7.40	6.39	2	25	p=0.017
**HINT SRT**	19.99	6.61	12.60	54.80	16.19	3.52	11.00	26.20	p<0.0001
**HINT 1**	−3.37	1.29	−5.50	2.40	−4.02	0.75	−5.10	−1.80	p=0.004
**HINT 2**	−11.03	1.79	−13.8	−2.20	−11.78	1.02	−13.6	−9.40	p=0.012
**HINT 3**	−11.21	1.83	−14.7	−2.20	−11.73	1.13	−13.6	−9.40	p=0.140
**HINTC**	−7.26	1.27	−8.50	0.10	−7.94	0.83	−11.1	−5.90	p=0.001

*Note*. RGD 0.5. Random gap detection subtest 500 Hz. Scores are in milliseconds.

RGD 1. Random gap detection subtest 1000 Hz. Scores are in milliseconds.

RGD 2. Random gap detection subtest 2000 Hz. Scores are in milliseconds.

RGD 4. Random gap detection subtest 4000 Hz. Scores are in milliseconds.

RGD C. Random gap detection subtest clicks. Scores are in milliseconds.

HINT. Hearing-in-noise test. Scores are in dB signal-to-noise ratio. HINT SRT: binaural speech recognition threshold for sentences in quiet; HINT 1: noise and sentences delivered at 0° azimuth; HINT 2: noise delivered to the right ear (90° azimuth) and sentences delivered to the front (0° azimuth); HINT 3: noise delivered to the left ear (270° azimuth) and sentences delivered to the front (0° azimuth); HINT C: composite value calculated combining the previously three HINT conditions.

### Bivariate and multivariate regression analyses

Using simple (bivariate) linear regression analyses, the variables significantly associated with the mean air conduction hearing threshold (PTA, binaural average of audiometric hearing thresholds for 500–8000 Hz) were age, solvent exposure and years of factory work exposed to solvents. Variables significantly associated with the mean total TEOAE response amplitude (dB SNR) included gender, solvent exposure and years of factory work exposed to solvents. Variables significantly associated with RGD were age, solvent exposure, years of factory work exposed to solvents and past noise exposure. Variables significantly associated with HINT SRT were age, solvent exposure and years of factory work exposed to solvents. Finally, the variables significantly associated with HINT composite included age, solvent exposure and years of factory work exposed to solvents.

Using multiple linear regression analyses for each hearing outcome independently, the mean air conduction hearing threshold (PTA, binaural average of audiometric hearing thresholds for 500–8000 Hz), the mean total TEOAE response amplitude (dB SNR), mean score for RGD, mean score for HINT SRT, and mean value for HINT composite were not significantly predicted by smoking and shooting practice. As Table 4 shows, the variables remaining significantly associated with PTA in the final multivariate model were age and solvent exposure. The total response for TEOAE (500–5000 Hz, Table 5) was best predicted by alcohol consumption and solvent exposure. The mean score for RGD subtests (500, 1000, 2000, 4000 Hz, and clicks, Table 6) was best predicted by age, solvent exposure and past noise exposure. The mean score for HINT SRT (Table 7) was best predicted by age and solvent exposure. The mean value for HINT composite (Table 8) was best predicted only by solvent exposure.

**Table 4:** Bivariate and multivariate linear regression analysis for PTA (500 Hz-8000 Hz) outcome

**Bivariate model**				**Multivariate model**		**Final multivariate model**		
**Characteristic**		**Beta**	**p**	**Beta**	**p**	**Characteristic**	**Beta**	**p**
Age		0.411	p<0.0001	0.089	0.894	Age	0.365	p<0.0001
Male		0.056	0.594	−0.248	0.093			
**Solvent exp.**	EXP	0.332	0.001	−0.122	0.855	EXP	0.296	0.002
	NEXP	Reference						
Tenure		0.415	p<0.0001	−0.068	0.834			
**Risk factors**	Cigarettes	0.197	0.158	0.246	0.083			
	Alcohol	0.200	0.152	0.278	0.043			
	ShootWP	−0.060	0.635	0.040	0.754			
	ShootNP	−0.035	0.786	0.162	0.246			
	No Shoot	Reference						
	Pastnoise	0.174	0.095	0.117	0.377			
**Interactions**						**Interactions**		
Age*solvent				0.627	0.581			

*Note. ***Solvent exp**: EXP: solvent-exposed group, NEXP: non-exposed group (reference); **Tenure**: number of years working at the factory exposed to solvents; **Cigarettes**: Number of cigarettes smoked per week; **Alcohol**: Number of drinks per month; **Shoot WP**: history of shooting practice wearing hearing protection; **ShootNP**: history of shooting practice without wearing hearing protection; **No shoot**: absence of shooting practice (reference); **Pastnoise:** history of previous occupational noise exposure; Interaction Age*solvent: factor of age times solvent group (exposed versus non-exposed).

**Table 5:** Bivariate and multivariate linear regression analysis for TEOAE outcome

**Bivariate model**				**Multivariate model**		**Final multivariate model**		
**Characteristic**		**Beta**	**p**	**Beta**	**p**	**Characteristic**	**Beta**	**p**
Age		−0.130	0.256	0.213	0.786			
Male		−0.247	0.027	−0.091	0.605			
**Solvent exp**.	EXP	−0.317	0.004	−0.290	0.723	EXP	−0.421	0.005
	NEXP	Reference						
Tenure		−0.346	0.002	−0.535	0.183			
**Risk factors**	Cigarettes	−0.259	0.075	−0.033	0.849			
	Alcohol	−0.206	0.166	−0.342	0.058	Alcohol	−0.352	0.019
	ShootWP	0.028	0.835	0.119	0.437			
	ShootNP	0.053	0.695	0.192	0.254			
	No Shoot	Reference						
	Pastnoise	−0.176	0.121	−0.051	0.750			
**Interactions**						**Interactions**		
Age*solvent				0.180	0.895			

*Note. ***Solvent exp**: EXP: solvent-exposed group, NEXP: non-exposed group (reference); **Tenure**: number of years working at the factory exposed to solvents; **Cigarettes**: Number of cigarettes smoked per week; **Alcohol**: Number of drinks per month; **Shoot WP**: history of shooting practice wearing hearing protection; **ShootNP**: history of shooting practice without wearing hearing protection; **No shoot**: absence of shooting practice (reference); **Pastnoise:** history of previous occupational noise exposure; Interaction Age*solvent: factor of age times solvent group (exposed versus non-exposed).

**Table 6:** Bivariate and multivariate linear regression analysis for Random gap detection (average)

**Bivariate model**				**Multivariate model**		**Final multivariate model**		
**Characteristic**		**Beta**	**p**	**Beta**	**p**	**Characteristic**	**Beta**	**p**
Age		0.293	0.007	0.527	0.466	Age	0.255	0.008
Male		0.086	0.431	0.033	0.840			
**Solvent exp.**	EXP	0.403	p<0.0001	0.177	0.813	EXP	0.239	0.027
	NEXP	Reference						
Tenure		0.467	p<0.0001	0.082	0.821			
**Risk factors**	Cigarettes	−0.048	0.742	−0.255	0.098			
	Alcohol	−0.104	0.477	−0.031	0.834			
	ShootWP	−0.064	0.626	0.021	0.883			
	ShootNP	0.224	0.086	0.257	0.098			
	No Shoot	Reference						
	Pastnoise	0.427	p<0.0001	0.384	0.012	Pastnoise	0.303	0.005
**Interactions**						**Interactions**		
Age*solvent				−0.250	0.840			

*Note. ***Solvent exp**: EXP: solvent-exposed group, NEXP: non-exposed group (reference); **Tenure**: number of years working at the factory exposed to solvents; **Cigarettes**: Number of cigarettes smoked per week; **Alcohol**: Number of drinks per month; **Shoot WP**: history of shooting practice wearing hearing protection; **ShootNP**: history of shooting practice without wearing hearing protection; **No shoot**: absence of shooting practice (reference); **Pastnoise:** history of previous occupational noise exposure; Interaction Age*solvent: factor of age times solvent group (exposed versus non-exposed).

**Table 7:** Bivariate and multivariate linear regression analysis for HINT SRT outcome

**Bivariate Model**				**Multivariate model**		**Final multivariate model**		
**Characteristic**		**Beta**	**p**	**Beta**	**P**	**Characteristic**	**Beta**	**p**
Age		0.317	0.003	−0.080	0.915	Age	0.252	0.014
Male		0.201	0.061	0.006	0.973			
**Solvent exp.**	EXP	0.343	0.001	−0.176	0.826	EXP	0.330	0.001
	NEXP	Reference						
Tenure		0.370	p<0.0001	0.010	0.978			
**Risk factors**	Cigarettes	0.089	0.542	0.204	0.204			
	Alcohol	0.207	0.159	0.220	0.184			
	ShootWP	0.052	0.698	0.013	0.932			
	ShootNP	−0.104	0.431	−0.039	0.810			
	No Shoot	Reference						
	Pastnoise	−0.011	0.918	−0.287	0.065			
**Interactions**						**Interactions**		
Age*solvent				0.567	0.669			

*Note. ***Solvent exp**: EXP: solvent-exposed group, NEXP: non-exposed group (reference); **Tenure**: number of years working at the factory exposed to solvents; **Cigarettes**: Number of cigarettes smoked per week; **Alcohol**: Number of drinks per month; **Shoot WP**: history of shooting practice wearing hearing protection; **ShootNP**: history of shooting practice without wearing hearing protection; **No shoot**: absence of shooting practice (reference); **Pastnoise:** history of previous occupational noise exposure; Interaction Age*solvent: factor of age times solvent group (exposed versus non-exposed).

**Table 8:** Bivariate and multivariate linear regression analysis for HINT composite outcome

**Bivariate model**				**Multivariate model**		**Final multivariate model**		
**Characteristic**		**Beta**	**p**	**Beta**	**p**	**Characteristic**	**Beta**	**p**
Age		0.250	0.020	0.745	0.289			
Male		0.092	0.392	0.147	0.361			
**Solvent exp.**	EXP	0.306	0.004	1.013	0.177	EXP	0.306	0.004
	NEXP	Reference						
Tenure		0.263	0.013	−0.231	0.515			
**Risk factors**	Cigarettes	0.065	0.660	0.116	0.434			
	Alcohol	−0.051	0.732	−0.124	0.413			
	ShootWP	−0.062	0.640	−0.101	0.462			
	ShootNP	−0.057	0.670	−0.003	0.982			
	No Shoot	Reference						
	Pastnoise	0.098	0.368	0.078	0.534			
**Interactions**						**Interactions**		
Age*solvent				−0.764	0.534			

*Note. ***Solvent exp**: EXP: solvent-exposed group, NEXP: non-exposed group (reference); **Tenure**: number of years working at the factory exposed to solvents; **Cigarettes**: Number of cigarettes smoked per week; **Alcohol**: Number of drinks per month; **Shoot WP**: history of shooting practice wearing hearing protection; **ShootNP**: history of shooting practice without wearing hearing protection; **No shoot**: absence of shooting practice (reference); **Pastnoise:** history of previous occupational noise exposure; Interaction Age*solvent: factor of age times solvent group (exposed versus non-exposed).

## Discussion

### Effects of solvents on pure-tone thresholds

Solvent-exposed subjects presented with poorer hearing thresholds than non-exposed control subjects for the mid to high frequency region (1000, 2000 and 3000 Hz in the right ear, and 1000, 2000, 3000 and 8000 Hz in the left ear). In the bivariate models solvent exposure was significantly associated with the binaural average of hearing thresholds (500–8000 Hz). Similarly, age and years of factory work exposed to solvents were significantly associated with this auditory outcome. In the final multivariate model, only age and solvent exposure were significant predictors of the binaural average of hearing thresholds (500–8000 Hz). All these results suggest that solvents, even in the absence of excessive noise exposure, have a deleterious effect on pure-tone thresholds. This finding is in agreement with previous human and animal research evidence (see Fuente & McPherson [27] for review). Regarding the frequency region affected by solvents, previous studies have found an association between poorer pure-tone thresholds for the mid-and high-frequency range of audiometric frequencies and solvents such as styrene [28]. Sliwinska-Kowalska et al. [29] reported significantly higher mean audiometric thresholds for a styrene-exposed group of workers at 2, 4, and 6 kHz when compared to a noise-only and an unexposed control group. Johnson et al. [13] reported poorer pure-tone thresholds in the high frequency range (3–8 kHz) for a group of noise-and styrene-exposed workers than non-exposed workers. Similarly, Fuente et al. [11] in a group of workers exposed to a mixture of solvents and with noise exposure below 85 dBA, found a significant association between solvent exposure and the average of pure-tone thresholds between 3000–6000 Hz, which was the range of hearing thresholds included in this multivariate analysis. Chang et al. [30] found among toluene-and noise-exposed subjects worse hearing thresholds for various frequencies, including 500 Hz. Thus, based on the results of this study and on data from previous research, a broad range of audiometric frequencies are affected by solvent exposure in humans. Animal models have found that solvents, including styrene and toluene, induce cochlear damage in the mid-to high-frequency range which is similar to the findings of this study and most of the studies discussed above.

Age was expected to appear as a significant predictor for hearing thresholds in this study, as it has been extensively documented that this variable is associated with hearing threshold level [31,32].

### Effects of solvents on TOAEs

One of the aims of the present research was to investigate the added value of OAEs for the detection of SIHL. Solvent-exposed subjects presented with significantly lower dB SNR TEOAE responses for the right and left ear as compared to non-exposed control subjects. In the bivariate models gender (male), solvent exposure and years of factory work exposed to solvents were significantly associated with TEOAEs. The multivariate analysis for TEOAEs showed that alcohol consumption and solvent exposure were the only two variables that remained significantly associated with this hearing outcome. This indicates the potential ototoxic properties of solvents in humans and is in agreement with data obtained from animal models [4,5,33]. It has been suggested from animal models that SIHL is associated with OHC damage. However, from human studies there has still been limited evidence on cochlear dysfunction associated with SIHL. Sulkowski et al. [12] found lower amplitudes for TEOAE and distortion product otoacoustic emissions (DPOAE) among solvent-exposed subjects in comparison to non-exposed control subjects. Johnson et al. [13] did not find a significant association between solvent exposure and DPOAE signal level. However, at lower signal levels the input/output function of the DPOAEs styrene-exposed subjects presented with lower amplitudes than controls and subjects only exposed to noise. Therefore, the results of this research provide further evidence that solvents may adversely affect the OHC in human subjects.

Alcohol consumption was a significant predictor for TEAOE results only in the multivariate regression model when accompanied with solvent exposure. This suggests that alcohol consumption may interact with solvent exposure to induce OHC dysfunction. Previous research has demonstrated that acute alcohol consumption may induce a temporary reduction in DPOAE amplitudes at high frequencies without affecting auditory thresholds [34].

It was not surprising that gender appeared to be significantly associated with TEOAEs in the bivariate model. Previous research has demonstrated that female subjects have higher otoacoustic emission amplitudes than male subjects [35,36]. In the present research females obtained higher TEOAE amplitudes as compared to males.

Based on the results discussed above, we may conclude that at least part of the decrement in hearing thresholds among solvent-exposed subjects relates to OHC dysfunction, and that solvent-exposure is associated with OHC dysfunction in human subjects. This suggests that subjects exposed to solvents in this study may have presented with early peripheral auditory dysfunction.

### Effects of solvents on the central auditory system

Solvent-exposed subjects presented with significantly poorer RGD results than non-exposed control subjects for all RGD subtests. The bivariate models showed that age, solvent exposure, history of past occupational noise exposure and years of factory work exposed to solvents were significantly associated with this auditory outcome. The multivariate analysis showed that age, history of past occupational noise exposure and solvent exposure were significant predictors of the score obtained in the RGD assessment. RGD evaluates temporal resolution in the auditory system. The results of this study suggest therefore that solvent exposure is associated with an adverse effect on temporal resolution abilities in human subjects. Temporal resolution relates to the capacity of the auditory fibres to encode timing information. Age was expected to be associated with RGD, as biological-based changes in the central auditory system have been reported [37], and the RGD relies on the synchrony of the auditory fibres.

Only a few studies investigating temporal resolution in solvent-exposed subjects have been carried out. Zamyslowska-Szmytke et al. [18] did not find an association between styrene exposure and performance in a temporal resolution task, the gaps-in-noise test (GIN [38]). Differences between the present study results and those obtained by Zamyslowska-Szmytke et al. may be due to the difference in solvent exposures. In the present study subjects from both paint-making factories were exposed to a mixture of solvents comprising xylene, toluene, methyl ethyl ketone, and Stoddard solvent whereas in the study of Zamyslowska-Szmytke et al. subjects working in the fibreglass industry were mainly exposed to styrene. Thus, we may hypothesise that different solvents induce different auditory signs and symptoms at the level of CANS. However, another aspect that should also be considered is the type of task used to assess temporal resolution. The GIN test assesses temporal resolution based on a task of gap detection in an ongoing stimulus of white noise. The RGD assesses temporal resolution based on a task where the subject is required to identify the gap in the onsets of two equal stimuli (tone bursts or clicks). Consequently, differences between both studies may also be due to the different procedures used to investigate temporal resolution. Further research using both procedures—GIN and RGD—among subjects exposed to mixtures of solvents or isolated solvents should be conducted, to clarify the differences observed between these studies.

### Effects of solvents on speech perception

The bivariate models showed that age, solvent exposure and years of factory work exposed to solvents were significantly associated with HINT SRT and HINT composite. The multivariate regression analysis for HINT SRT showed that age and solvent exposure were significant predictors for this auditory outcome. For HINT composite the multivariate regression analysis showed that solvent exposure was the only significant predictor for this auditory outcome. Thus, for both speech perception in quiet and speech perception in noise, solvent exposure appeared significantly associated. This is in agreement with previous studies which have shown that solvent-exposed workers presented with poor results for speech-in-noise tasks [13,39]. We hypothesise that the effect of solvents on speech perception abilities may be due to the effects of solvents on pure-tone thresholds along with adverse effects on the central auditory nervous system. Speech perception, especially in difficult listening environments, is a complex hearing function that relies not only on adequate sound detection but also on normal processing of timing information within the CANS [40,41]. As discussed above, solvent-exposed subjects in this study presented with poorer performance than non-exposed subjects on a task based on temporal processing. Therefore, speech perception may be affected in solvent-exposed persons as a secondary effect of solvents on the peripheral and central auditory systems. Taking into consideration the importance of speech perception in daily life activities, it is expected that solvent-exposed persons may encounter difficulties in a number of listening activities and thus their quality of life may be adversely affected. Further research should be carried out to determine the impact of solvent exposure on persons’ quality of life.

### Limitations of the study

The present study has some limitations. Partial exposure data was the main limitation in the characterisation of the solvent-exposed group. Airborne solvent concentrations were only available from the past three years. In terms of exposure history, we could only obtain the number of years each worker was exposed to solvents either in the current job category, or performing other duties in the same factory or at another paint-making factory. Therefore, accurate calculation of working life exposure indexes could not be obtained. It is most likely that exposure levels as well as the components of the solvent mixtures have changed throughout the working life of the workers. The lack of these data limits the results of the present study, and associations between levels of solvent exposure and auditory function could not be investigated. Another limitation was the selection of the control group. This group came from a totally different industrial sector to the solvent-exposed subjects. It was decided not to select control subjects from the paint-making factories studied as these subjects may still have some levels of exposure to solvents. Administrative personnel usually have to visit the manufacturing area and thus solvent exposure still occurs. Non-exposed control group subjects were employees of the University of Chile who performed different duties, from cleaning to administration. Thus, having a control group from a different area of industry may have partially biased the results found in the present study. However, it should be taken into account that even if solvent-exposed and control subjects came from different sectors of industry, they were matched according to their educational level and that may have diminished possible social differences that could affect test results.

### The use of different tests to identify SIHL

This study has shown that solvent exposure is associated with poorer sound detection thresholds, lower OAE response amplitudes, reduced central auditory functioning as measured by a temporal resolution task (RGD), and poorer speech perception abilities. These signs are likely to be due to the oto- and neurotoxic properties of solvents. Therefore, the assessment of SIHL should include tests exploring the peripheral and central auditory system. Evidence from this and other studies has consistently demonstrated that the exclusive use of pure-tone audiometry is not adequate when assessing SIHL. A conference on the deleterious effects of noise and solvents on the auditory system was held in Lodz, Poland [42]. Among the suggestions that arose from this conference, the need to include central auditory tests in the monitoring of SIHL was highlighted. The present study provides evidence that the RGD test and the HINT are suitable clinical tools for the detection of central auditory dysfunction associated with solvent exposure. Further research investigating central auditory effects using electrophysiological measurements should also be conducted. Finally, this study provides evidence concerning the use of otoacoustic emissions to detect cochlear-related dysfunction induced by solvent exposure. This tool should be incorporated in hearing conservation programmes targeted at solvent-exposed workers with the aim to early identify deleterious effects of solvents on the OHC. In conclusion, according to the results of this research pure-tone audiometry along with TEOAEs and the RGD test are suitable procedures to evaluate SIHL. If time is available and the costs are considered reasonable the HINT can be also incorporated in the test battery to determine the impact of SIHL on speech perception abilities.

## Conclusions

According to the findings of this research solvent-exposure is associated with both cochlear and central auditory dysfunction. Test results for PTA, TEOAE, RGD and HINT appeared significantly associated with solvent exposure. Therefore, a comprehensive audiological test battery which incorporates peripheral and central tests should be used when assessing solvent-exposed individuals. The tests utilised in the present research, or similar ones, can be used for such purpose. In particular, the RGD test appears as a sensitive tool to detect central auditory dysfunction. Finally, from the results of this research we can conclude that the sole use of pure-tone audiometry is not sufficient to monitor and/or assess hearing among solvent-exposed workers and that solvent-exposed workers should be incorporated in hearing conservation programmes regardless of their noise exposure levels.

## Competing interests

The authors declare that they have no competing interests.

## Authors’ contributions

AF contributed to the study design, collection, analysis of data, and writing the paper. BM contributed to the study design, analysis of data and writing the paper. LH contributed to the analysis of data and writing the paper. All authors approved the final version of the manuscript.

## Pre-publication history

The pre-publication history for this paper can be accessed here:

http://www.biomedcentral.com/1471-2458/13/39/prepub
